# Costing Interventions for Developing an Essential Package of Health Services: Application of a Rapid Method and Results From Pakistan

**DOI:** 10.34172/ijhpm.2023.8006

**Published:** 2024-01-07

**Authors:** Wajeeha Raza, Wahaj Zulfiqar, Mashal Murad Shah, Maryam Huda, Syeda Shehirbano Akhtar, Urooj Aqeel, Saira Kanwal, Muhammad Khalid, Raza Zaidi, Maarten Jansen, Nichola Kitson, Leon Bijlmakers, Sameen Siddiqi, Ala Alwan, Anna Vassall, Sergio Torres-Rueda

**Affiliations:** ^1^Centre for Health Economics, University of York, York, UK; ^2^Ministry of National Health Services, Regulations and Coordination, Islamabad, Pakistan; ^3^Department of Community Health Sciences, Aga Khan University, Karachi, Pakistan; ^4^Department of Health Evidence, Radboud Institute of Health Sciences, Radboud University Medical Centre, Nijmegen, The Netherlands; ^5^Department of Global Health & Development, London School of Hygiene and Tropical Medicine, London, UK; ^6^DCP3 Country Translation Project, London School of Hygiene and Tropical Medicine, London, UK

**Keywords:** Costing, UHC, Benefit Package, Pakistan

## Abstract

**Background:** The Federal Ministry of National Health Services, Regulations and Coordination (MNHSR&C) in Pakistan has committed to progress towards universal health coverage (UHC) by 2030 by providing an Essential Package of Health Services (EPHS). Starting in 2019, the Disease Control Priorities 3rd edition (DCP3) evidence framework was used to guide the development of Pakistan’s EPHS. In this paper, we describe the methods and results of a rapid costing approach used to inform the EPHS design process.

**Methods:** A total of 167 unit costs were calculated through a context-specific, normative, ingredients-based, and bottom-up economic costing approach. Costs were constructed by determining resource use from descriptions provided by MNHSR&C and validated by technical experts. Price data from publicly available sources were used. Deterministic univariate sensitivity analyses were carried out.

**Results:** Unit costs ranged from 2019 US$ 0.27 to 2019 US$ 1478. Interventions in the cancer package of services had the highest average cost (2019 US$ 837) while interventions in the environmental package of services had the lowest (2019 US$ 0.68). Cost drivers varied by platform; the two largest drivers were drug regimens and surgery-related costs. Sensitivity analyses suggest our results are not sensitive to changes in staff salary but are sensitive to changes in medicine pricing.

**Conclusion:** We estimated a large number of context-specific unit costs, over a six-month period, demonstrating a rapid costing method suitable for EPHS design.

## Background

**Key Messages**

**Implications for policy makers**

- This paper demonstrates a rapid method to calculate context-specific and comparable unit costs which was successfully used in the Essential Package of Health Services (EPHS) design process in Pakistan.
- We report the results for the first comprehensive dataset of unit costs for 167 health interventions based on localised evidence in Pakistan.
- Our method is transparent and unit cost estimates are disaggregated by input type, highlighting the cost drivers for each intervention. Our method can be replicated for future priority setting exercises in Pakistan as well as used in other contexts.

**Implications for the public**

This paper provides details of the methodology and the results of the first rapid health systems-wide costing exercise in Pakistan. The calculation of local costs was an important component of the Essential Package of Health Services (EPHS) design process in Pakistan. Policy-makers used this information on costs, along with other evidence, to design a package of services that is effective, efficient and affordable. The costs presented here can also be used by policy-makers to consider the expansion of existing services or the inclusion of new services. The approaches developed to carry out this work could be used to improve decisions around healthcare service prioritisation in other countries.

Countries around the world have strengthened their commitment to universal health coverage (UHC) in recent years. UHC has been enshrined in Sustainable Development Goal target 3.8 and calls for access to quality essential healthcare services for all.^1^ There are many types of health services that countries could potentially deliver, but budgetary constraints force policy-makers to limit the number and coverage of interventions financed through public expenditure. In order to set health sector priorities, many countries have embarked on designing or revising essential package of health services (EPHS). This approach allows for explicit system-wide priority setting within a given budget envelope.^2^

 Pakistan’s commitment to providing a UHC EPHS was stated in its 12th Five-Year Plan (2018-2023) and National Action Plan (2019-2023) for the health sector.^3^ The Federal Ministry of National Health Services, Regulations and Coordination (MNHSR&C) decided to use the Disease Control Priorities 3rd edition (DCP3) as a starting point and framework of reference for the process of priority setting of health services provided by the public sector at the district level.^4^ DCP3 is a multi-year project that sought to synthesise global evidence of cost and cost-effectiveness evidence across disease areas, and provides the evidence base for an essential universal health coverage (EUHC) package composed of 218 interventions as a guide for low- and middle-income countries (LMICs).^5^

 In 2019, the MNHSR&C, jointly with the provincial departments of health and key stakeholders, compared the current scope of essential health services offered in Pakistan against the services covered by the EUHC. They recommended that a subset of EUHC interventions should be assessed for inclusion in Pakistan’s UHC-EPHS.^6^ This paper reports on the costing of this subset of DCP3 interventions as part of a broader process of UHC-EPHS design in Pakistan.

 This paper has two objectives: (*i*) to demonstrate a rapid costing methodology that can be used in the process of estimating unit costs for EPHS design in LMICs, and (*ii*) to present the first comprehensive dataset of unit costs for health interventions based on localised evidence in Pakistan. These unit costs are the building blocks needed to estimate the package’s total cost, the relative budget impact of individual interventions and the affordability of the EPHS given available fiscal space, which are key analytical components in the EPHS design process.

## Methods

 The general costing approach presented here was designed during a meeting with national stakeholders convened by the Health Planning, System Strengthening & Information Analysis Unit (HPSIU) of the MNHSR&C in Islamabad in July 2019.

 Ideally, cost estimation for a UHC EPHS design would rely on local primary data collection. However, we used secondary data sources for several reasons. Firstly, costing current service provision would likely reflect service delivery of low quality; as the EPHS aims to deliver high quality services, collecting primary data could lead to cost underestimation. Further, it was estimated that 135 (62%) of the 218 EUHC package interventions were not routinely available in Pakistan’s public sector.^6^ Lastly, primary data collection is a resource-intensive exercise which was not feasible in the project timeframe.

 Obtaining unit costs from the published global literature was also considered. However, a review of cost-effectiveness databases found a scarcity of high-quality costing estimates appropriate for Pakistan.^7,8^ Adapting and transferring cost data across settings can be misleading as information on resource use and prices (such as lengths of patient consultations, health worker salaries and prices of medications and equipment) varies greatly between countries.^9-11^ Further, variation in the context-specific service configuration can have affect costs and efficiency.^12^ Lastly, published data is often not sufficiently disaggregated and costing studies often employ different methodologies leading to evidence of varying levels of quality, a challenge when attempting comparability across many interventions.

 Consequently, in consultation with stakeholders, we instead opted to develop a context-specific, rapid normative method to estimate the cost of DCP3 interventions. The costs were estimated by a joint team from the Aga Khan University and the London School of Hygiene & Tropical Medicine, using data provided by the HPSIU and reviewed and validated by local technical experts. This rapid costing was conducted over a six-month period.

###  General Approach

 We carried out an ingredients-based costing and took an economic costing approached, meaning that we accounted for the value of all resources used, regardless of whether financial expenditure was expected. A bottom-up approach to costing using both secondary sources and expert opinion was applied. We assumed a provider’s perspective and used a one-year time horizon. Our approach followed the principles set in the Global Health Costing Consortium reference case,^13^ a gold standard for the costing of health interventions in LMICs.

 We costed interventions across all five DCP3 EUHC delivery platforms: community-level, health centre, first-level hospitals, referral hospitals, and population-level. However, the priority setting exercise focused on a district package of services; population-level interventions were therefore excluded from the main analysis as they are operated and implemented at the national level. See Supplementary file 1 for population-level unit cost estimates and further information.

 The DCP3 EUHC contains 218 interventions.^5^ Following a preliminary review carried out by MNHSR&C, as well as consultations with provincial-level stakeholders and within HPSIU, 47 interventions were eliminated as not deemed immediately relevant to Pakistan. Further, 12 population-level interventions were excluded as not deemed implementable at the district level. Of the remaining 159 interventions, nine were broken down into multiple interventions because either the scope of the EUHC interventions were deemed to be too broad to assess, or the intervention could be delivered in multiple platforms. Consequently, a final shortlist of 170 Pakistan-adapted interventions was recommended for formal assessment and appraisal. Of these 170 interventions, three interventions were not costed as resource mapping was considered unfeasible (See Supplementary file 2). Consequently, 167 unit costs were calculated.

**Figure 1:**
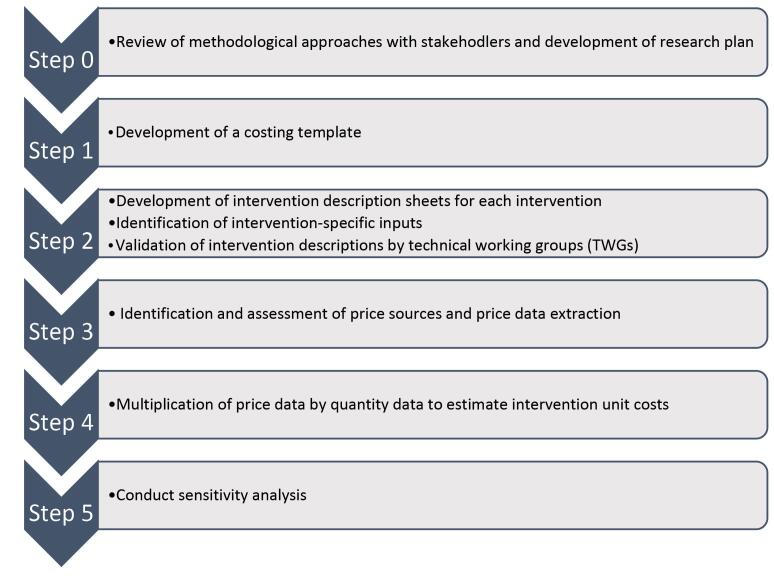
Steps for Costing Essential Package of Health Services in Pakistan.

 The costing approach comprised several steps as specified in Figure 1: (1) development of a costing template, (2) development of intervention description sheets for each intervention, identification of intervention-specific inputs and validation by technical working groups (TWGs), (3) identification and assessment of price sources and price data extraction, (4) combination of price and resource use data, and (5) sensitivity analysis. We calculated unit costs by estimating the resources needed and relevant prices per beneficiary per year (eg, cost per person treated for hypertension over a year). Costs were estimated in Pakistani rupees and converted to 2019 US dollars at an exchange rate of 155 PKR:USD.^14^

###  Costing Template

 The stakeholders felt that a cost estimation tool for EPHS design needed to show transparency, flexibility and ease of use, and should work across multiple platforms in the health system. As a result, the team designed a semi-automated user-friendly costing template in Microsoft® Excel. The template separated resource use data and prices and divided costs by input category. It allowed calculations of multiple unit costs per intervention (eg, interventions carried out in multiple platforms or by multiple types of health workers). It also granted the flexibility of entering multiple price lists per input.

###  Determining Resource Use

 To capture all resources used for each individual intervention, the HPSIU prepared intervention-specific description sheets detailing inputs necessary for service delivery: staff requirements (staff type and time), drug regimens, laboratory-based diagnostics, radiology, other supplies, and equipment per patient/year. These descriptions were developed based on the latest existing national guidelines (or, in their absence, international guidelines) for each intervention and expert opinion from senior Pakistani clinicians. For hospital-based interventions, the average number of inpatient bed-days, and whether the intervention involved surgery, were also specified by senior clinicians. The clinicians were identified by the primary academic partner, Aga Khan University, and HPSIU. Feedback from the clinicians was collected during two rounds of 3-day workshops. The different inputs needed for inpatient bed-days and surgeries were obtained from published sources.^15,16^

 The first draft of the intervention description sheets was compiled by the HPSIU and revised and amended by experts during several rounds of disease-specific TWG consultations. Final intervention descriptions were used for mapping individual interventions and compiling a list of resources used and quantities.^17^ Further details on resource use quantification can be found in Supplementary file 3.

 We only accounted for direct resource use in service provision. We did not include any indirect costs, above-service delivery costs or other overheads. We also do not include health system costs such as the cost of governance at the district level. Indirect costs were accounted for and included in a later stage of the design process. See Torres-Rueda et al for further details.^18^

###  Determining Prices

 A variety of price sources were available for each input. An assessment of strengths and weaknesses of different price sources was conducted. The quality of sources was assessed using three main criteria shown in Table 1, namely how recently the source had been published, whether it referred to the public or private sector, and whether it was applicable across settings within Pakistan. A hierarchy of sources was then established.

**Table 1:** Hierarchy of Price Sources

**Criteria**	**Rationale**
Is the price recent?	Up to date prices account for changes in inflation and are more relevant to the decision problem.
Is the source of price a public source?	This costing exercise uses a provider perspective for the public sector. The prices for inputs purchased by the public sector such as medicines and equipment can often differ substantially from private sector prices. We therefore attempted to use the purchase prices for public sector providers.
Is the source nationally representative?	At this stage, the UHC benefit package and its unit costs are a national exercise therefore the price sources used were ranked higher if they were nationally representative.

Abbreviation: UHC, universal health coverage.

 Details on price sources assessed and used can be found in Supplementary file 4. In summary, federal-level healthcare worker pay scales were used to determine average staff time pricing per health worker cadre.^19^ The primary source for medication price data used was the Sindh Health Department Procurement Price list of 2018-2019,^20^ as it was both recent and listed public sector prices. The first choice for supplies and equipment was a list of procurement prices from the Medical Emergency Resilience Fund 2019-2020.^21^ Building prices were obtained from Federal budgets (spaces)^22^ and a costing study carried out in Khyber Pakhtunkhwa province (utilities).^23^ A generic cost of furniture was added (10% of the cost of space).^24^ We were unable to construct diagnostic and radiology costs through an ingredients-based approach due to time constraints and the complexity of supplies and equipment used. We used the ‘Costing and Pricing of Services in Private Hospitals of Lahore: Summary Report’ as our primary source for diagnostic prices as it also used an ingredients-based approach consistent with our methodology.^25^ Generic prices for surgery and inpatient bed-day were obtained from the same sources as the resource use data.^15,16^

###  Published Unit Costs and Prices

 In nine instances, unit costs or the price of the main input of an intervention were obtained from the peer-reviewed literature. These alternative options were used when the unit cost or main price estimate was grossly out of line with available global evidence and no Pakistan-specific reason for the discrepancy could be identified after consultation with health economists knowledgeable of the specific area (See Supplementary file 5).

###  Sensitivity Analysis

 We carried out deterministic univariate sensitivity analyses for two key parameters. Staff salaries vary by province in Pakistan but, in our analysis, we used federal pay scale salaries for our base case unit costs (which are used in Islamabad Capital Territory and Baluchistan province). We carried out a sensitivity analysis using pay-scales for Sindh and Khyber Pakhtunkhwa provinces, estimating percentage changes in average intervention costs per platform. We also examined the sensitivity of unit costs to different medicine prices for the ten most costly interventions. Using the different medicine price sources reviewed, we recalculated unit costs applying the lowest and highest medicine costs available and present those ranges in relation to the base case unit costs.

## Results

 There is a high variation in the unit costs for interventions, ranging from 2019 US$ 0.27 to 2019 US$ 1477.76 as shown in Table 2. Interventions with the top five highest unit costs were all delivered in the referral hospital platform: treatment of early-stage childhood cancers (2019 US$ 1477.76), treatment of early-stage breast cancer (2019 US$ 1304.04), management of refractory illness (2019 US$ 673.43), repair of cleft lip and cleft palate (2019 US$ 515.11), and specialized tuberculosis services, including management of drug resistant tuberculosis (2019 US$ 493.21). Interventions with the five lowest unit costs were all delivered through the community-based platform: screening of hypertensive disorders in pregnancy (2019 US$ 0.27), providing guidance on early symptoms during emerging infectious outbreaks (2019 US$ 0.28), adolescent-friendly health service provision (2019 US$ 0.33), antenatal and post-partum education (2019 US$ 0.34) and larviciding and water management in high malaria transmission settings (2019 US$ 0.41).

**Table 2:** Unit Costs of Disease Control Priorities 3rd Edition Interventions in Pakistan by Platform and by Cluster (2019 US$)

**Platform**	**Cluster **	**Package**	**DCP Code**	**Intervention Name**	** Unit Cost (2019 US$) **
Community	RMNCH	Maternal and new-born health	C1	Antenatal and postpartum education on family planning	$0.34
Community	RMNCH	Maternal and new-born health	C2	Counselling of mothers on providing thermal care for preterm new-borns (delayed bath and skin to skin contact)	$0.47
Community	RMNCH	Maternal and new-born health	C3a	Management of labour and delivery in low-risk women by skilled attendant	$14.46
Community	RMNCH	Maternal and new-born health	C3b	Basic neonatal resuscitation following delivery	$1.01
Community	RMNCH	Maternal and new-born health	C4	Promotion of breastfeeding or complementary feeding by lay health workers	$0.71
Community	RMNCH	Child health	C8	Detection and management of severe acute malnutrition and referral in the presence of complications	$12.53
Community	RMNCH	Child health	C9	Detection and treatment of childhood infections (iCCM), including referral if danger signs	$0.52
Community	RMNCH	Child health	C10	Education on handwashing and safe disposal of children’s stools	$0.74
Community	RMNCH	Child health	C11	Pneumococcus vaccination	$11.46
Community	RMNCH	Child health	C12	Rotavirus vaccination	$5.66
Community	RMNCH	Child health	C14	Provision of vitamin A and zinc supplementation to children according to WHO guidelines, and provision of food supplementation to women and children in food insecure households	$13.00
Community	RMNCH	Child health	C16	Childhood vaccination series (diphtheria, pertussis, tetanus, polio, BCG, measles, hepatitis B, HiB, and rubella)	$11.67
Community	RMNCH	Child health	C17	In high malaria transmission settings, IRS in selected areas with high transmission and entomologic data on IRS susceptibility	$1.05
Community	RMNCH	School-age health	C18	Education of school children on oral health	$0.84
Community	RMNCH	School-age health	C19	Vision pre-screening by teachers; vision tests and provision of ready-made glasses on-site by eye specialists	$1.84
Community	RMNCH	School-age health	C20	School based HPV vaccination for girls (Also included in RH, HIV and cancer packages of services)	$26.11
Community	RMNCH	School-age health	C21	Mass drug administration for lymphatic filariasis, onchocerciasis, schistosomiasis, soil-transmitted helminthiases and trachoma, and food borne trematode infections (Also included in NTDs package of services)	$1.76
Community	RMNCH	Adolescent health	C23	Adolescent-friendly health services including provision of condoms to prevent STIs; provision of reversible contraception; treatment of injury in general and abuse in particular; and screening and treatment for STIs	$0.33
Community	RMNCH	Adolescent health	C24	Life skills training in schools to build social and emotional competencies	$4.47
Community	RMNCH	Reproductive health	C27a	Provision of iron and folic acid supplementation to pregnant women, and provision of food or caloric supplementation to pregnant women in food-insecure households	$35.40
Community	Infectious diseases	HIV	C28	Community-based HIV testing and counselling (for example, mobile units and venue-based testing), with appropriate referral or linkage to care and immediate initiation of lifelong ART	$1.40
Community	Infectious diseases	HIV	C30a	Provision of condoms	$14.15
Community	Infectious diseases	HIV	C30b	Provision of disposable syringes	$5.03
Community	Infectious diseases	TB	C32	Routine contact tracing to identify individuals exposed to TB and link them to care	$8.67
Community	Infectious diseases	Adult febrile illness	C34	Conduct larviciding and water-management programs in high malaria transmission areas where mosquito breeding sites can be identified and regularly targeted	$0.41
Community	Infectious diseases	Adult febrile illness	C41	Mass drug administration in low malaria transmission settings (including high-risk groups in geographic or demographic clusters)	$0.84
Community	Infectious diseases	NTDs	C43	Early detection and treatment of Chagas disease, human African trypanosomiasis, leprosy, and leishmaniases	$7.94
Community	Infectious diseases	Pandemics	C45	Identify and refer patients with high risk including pregnant women, young children, and those with underlying medical conditions	$0.56
Community	Infectious diseases	Pandemics	C46	In the context of an emerging infectious outbreak, provide advice and guidance on how to recognize early symptoms and signs and when to seek medical attention	$0.28
Community	NCIP	CVD	C47	Exercise-based pulmonary rehabilitation for patients with obstructive lung disease	$2.01
Community	NCIP	Mental health	C48	Self-managed treatment of migraine	$0.42
Community	NCIP	Environmental	C51	WASH behaviour change interventions, such as community-led total sanitation	$0.68
Community	Health services	Rehabilitation	C53a	Identification of ECD rehabilitation interventions	$0.75
Community	Health services	Rehabilitation	C56	Pressure area prevention and supportive seating interventions for wheelchair users	$0.41
Community	RMNCH	Maternal and new-born health	HC4a	Condoms and hormonal contraceptives	$9.50
Community	RMNCH	Maternal and new-born health	HC5a	Counselling on kangaroo care for new-borns	$0.42
Community	RMNCH	Maternal and new-born health	HC9a	Screening of hypertensive disorders in pregnancy	$0.27
Community	Infectious diseases	TB	HC28	Screening for HIV in all individuals with a diagnosis of active TB; if HIV infection is present, start (or refer for) ARV treatment and HIV care	$1.53
Community	NCIP	Palliative care	HC66	Psychosocial support and counselling services for individuals with serious, complex, or life-limiting health problems and their caregivers	$4.47
Community	Infectious diseases	TB	P5	Systematic identification of individuals with TB symptoms among high-risk groups and linkage to care (“active case finding”)	$0.49
Health Centre	RMNCH	Maternal and new-born health	C3c	Management of labour and delivery in low-risk women by skilled attendant	$14.94
Health Centre	RMNCH	Maternal and new-born health	C3d	Basic neonatal resuscitation following delivery	$1.09
Health Centre	Infectious diseases	Adult febrile illness	C33	For malaria due to *Plasmodium vivax*, test for G6PD deficiency; if normal, add chloroquine or chloroquine plus 14-day course of primaquine	$1.65
Health Centre	RMNCH	Maternal and new-born health	C5	Tetanus toxoid immunization among schoolchildren and among women attending antenatal care	$0.67
Health Centre	RMNCH	Reproductive health	C27b	Provision of iron and folic acid supplementation to pregnant women, and provision of food or caloric supplementation to pregnant women in food-insecure households	$35.68
Health Centre	Health services	Rehabilitation	C53b	ECD rehabilitation interventions	$8.10
Health Centre	RMNCH	Maternal and new-born health	HC1	Early detection and treatment of neonatal pneumonia with oral antibiotics	$3.41
Health Centre	RMNCH	Maternal and new-born health	HC2	Management of miscarriage or incomplete abortion and post abortion care	$18.09
Health Centre	RMNCH	Maternal and new-born health	HC3	Management of preterm premature rupture of membranes, including administration of antibiotics	$110.28
Health Centre	RMNCH	Maternal and new-born health	HC4b	Condoms and hormonal contraceptives	$9.50
Health Centre	RMNCH	Maternal and new-born health	HC5b	Counselling on kangaroo care for new-borns	$0.42
Health Centre	RMNCH	Maternal and new-born health	HC7	Pharmacological termination of pregnancy	$10.74
Health Centre	RMNCH	Maternal and new-born health	HC9b	Screening and management of hypertensive disorders in pregnancy	$4.44
Health Centre	RMNCH	Maternal and new-born health	HC11	Management of labour and delivery in low-risk women (BEmNOC), including initial treatment of obstetric or delivery complications prior to transfer	$19.92
Health Centre	RMNCH	Child health	HC12	Detection and treatment of childhood infections with danger signs (IMCI)	$4.66
Health Centre	RMNCH	Adolescent health	HC14	Psychological treatment for mood, anxiety, ADHD and disruptive behaviour disorders in adolescents	$1.28
Health Centre	RMNCH	Reproductive health	HC16	Post-gender-based violence care, including counselling, provision of emergency contraception, and rape-response referral (medical and judicial)	$9.40
Health Centre	RMNCH	Reproductive health	HC17	Syndromic management of common sexual and reproductive tract infections (for example, urethral discharge, genital ulcer, and others) according to WHO guidelines	$3.19
Health Centre	Infectious diseases	HIV	HC20	Hepatitis B and C testing of individuals identified in the national testing policy (based on endemicity and risk level), with appropriate referral of positive individuals to trained providers	$2.43
Health Centre	Infectious diseases	HIV	HC21	Partner notification and expedited treatment for common STIs, including HIV	$2.31
Health Centre	Infectious diseases	HIV	HC23	Provider-initiated testing and counselling for HIV, STIs, and hepatitis for all in contact with the health system in high prevalence settings, including prenatal care with appropriate referral or linkage to care including immediate ART initiation for those testing positive for HIV	$2.73
Health Centre	Infectious diseases	HIV	HC25	Provision of voluntary medical male circumcision in settings with high prevalence of HIV	$25.03
Health Centre	Infectious diseases	TB	HC26	For PLHIV and children under five who are close contacts or household members of individuals with active TB, perform symptom screening and chest radiograph; if there is no active TB, provide isoniazid preventive therapy according to current WHO guidelines	$12.62
Health Centre	Infectious diseases	TB	HC27	Diagnosis of TB, including assessment of rifampicin resistance using rapid molecular diagnostics (UltraXpert), and initiation of first-line treatment per current WHO guidelines for drug susceptible TB; referral for confirmation, further assessment of drug resistance, and treatment of drug-resistant TB	$57.97
Health Centre	Infectious diseases	TB	HC29	Screening for latent TB infection following a new diagnosis of HIV, followed by yearly screening among PLHIV at high risk of TB exposure; initiation of isoniazid preventive therapy among all individuals who screen positive but do not have evidence of active TB	$9.79
Health Centre	Infectious diseases	Adult febrile illness	HC30	Evaluation and management of fever in clinically stable individuals using WHO IMAI guidelines, with referral of unstable individuals to first-level hospital care	$2.62
Health Centre	Infectious diseases	Adult febrile illness	HC32	Provision of insecticide-treated nets to children and pregnant women attending health centres	$5.36
Health Centre	Infectious diseases	Pandemics	HC33	Identify and refer to higher levels of healthcare patients with sings of progressive illness	$3.06
Health Centre	NCDIP	CVD	HC36	Long-term combination therapy for persons with multiple CVD risk factors, including screening for CVD in community settings using non-lab-based tools to assess overall CVD risk	$6.20
Health Centre	NCIP	CVD	HC37	Low-dose inhaled corticosteroids and bronchodilators for asthma and for selected patients with COPD	$1.59
Health Centre	NCIP	CVD	HC38	Provision of aspirin for all cases of suspected acute myocardial infarction	$0.56
Health Centre	NCIP	CVD	HC39a	Screening and management of albuminuric kidney disease with ACEi or ARBs, including targeted screening among people with diabetes	$6.29
Health Centre	NCIP	CVD	HC41	Secondary prophylaxis with penicillin for rheumatic fever or established rheumatic heart disease	$1.86
Health Centre	NCIP	CVD	HC42	Treatment of acute pharyngitis in children to prevent rheumatic fever	$3.04
Health Centre	NCIP	CVD	HC45	Opportunistic screening for hypertension for all adults and initiation of treatment among individuals with severe hypertension and/or multiple risk factors	$13.66
Health Centre	NCIP	CVD	HC46	Tobacco cessation counselling and use of nicotine replacement therapy in certain circumstances	$12.39
Health Centre	NCIP	Mental health	HC48	Interventions to support caregivers of patients with dementia	$5.43
Health Centre	NCIP	Mental health	HC49	Management of bipolar disorder using generic mood-stabilizing medications and psychosocial treatment	$56.89
Health Centre	NCIP	Mental health	HC50	Management of depression and anxiety disorders with psychological and generic antidepressant therapy	$20.36
Health Centre	NCIP	Mental health	HC53	Screening and brief intervention for alcohol use disorders	$7.98
Health Centre	NCIP	Musculoskeletal	HC55	Calcium and vitamin D supplementation for primary prevention of osteoporosis in high-risk individuals	$2.13
Health Centre	NCIP	Congenital disorders	HC56	Targeted screening for congenital hearing loss in high-risk children, using otoacoustic emissions testing	$8.76
Health Centre	Health services	Surgery	HC57a	Dental extraction	$12.11
Health Centre	Health services	Surgery	HC58a	Drainage of dental abscess	$9.10
Health Centre	Health services	Surgery	HC59	Drainage of superficial abscess	$10.02
Health Centre	Health services	Surgery	HC60	Management of non-displaced fractures	$8.42
Health Centre	Health services	Surgery	HC61	Resuscitation with basic life support measures	$1.03
Health Centre	Health services	Surgery	HC62	Suturing of lacerations	$1.77
Health Centre	Health services	Surgery	HC63a	Treatment of caries	$15.86
Health Centre	Health services	Rehabilitation	HC64	Basic management of musculoskeletal and neurological injuries and disorders, such as prescription of simple exercises and sling or cast provision	$5.48
Health Centre	Health services	Pathology	HC68	Health centre pathology services	$13.84
First-level hospital	NCIP	Injury	C50	Parent training of high-risk families, including nurse home visitation for child maltreatment	$1.93
First-level hospital	RMNCH	Maternal and new-born health	FLH1	Detection and management of foetal growth restriction	$321.46
First-level hospital	RMNCH	Maternal and new-born health	FLH2	Induction of labour post-term	$167.96
First-level hospital	RMNCH	Maternal and new-born health	FLH3	Jaundice Management of Phototherapy	$63.30
First-level hospital	RMNCH	Maternal and new-born health	FLH4	Management of eclampsia with magnesium sulphate, including initial stabilization at health centres	$106.79
First-level hospital	RMNCH	Maternal and new-born health	FLH5	Management of maternal sepsis, including early detection at health centres	$140.65
First-level hospital	RMNCH	Maternal and new-born health	FLH6	Management of new-born complications, neonatal meningitis, and other very serious infections requiring continuous supportive care (such as IV fluids and oxygen)	$79.06
First-level hospital	RMNCH	Maternal and new-born health	FLH7	Management of preterm labour with corticosteroids, including early detection at health centres	$157.66
First-level hospital	RMNCH	Maternal and new-born health	FLH8	Management of labour and delivery in high-risk women, including operative delivery (CEmONC)	$356.48
First-level hospital	RMNCH	Maternal and new-born health	FLH9	Surgery for ectopic pregnancy	$200.53
First-level hospital	RMNCH	Maternal and new-born health	FLH10	Surgical termination of pregnancy by manual vacuum aspiration and dilation and curettage	$115.24
First-level hospital	RMNCH	Child health	FLH11	Full supportive care for severe childhood infections with danger signs	$166.84
First-level hospital	RMNCH	Child health	FLH12	Management of severe acute malnutrition associated with serious infections	$150.08
First-level hospital	RMNCH	Reproductive health	FLH13	Early detection and treatment of early-stage cervical cancer	$170.42
First-level hospital	RMNCH	Reproductive health	FLH14	Insertion and removal of long-lasting contraceptives (IUCDs and implants)	$1.16
First-level hospital	RMNCH	Reproductive health	FLH15	Tubal ligation	$118.23
First-level hospital	RMNCH	Reproductive health	FLH16	Vasectomy	$115.60
First-level hospital	Infectious diseases	TB	FLH17	Referral of cases of treatment failure for drug susceptibility testing; enrolment of those with MDR-TB for treatment per WHO guidelines (either short- or long-term regimen)	$373.39
First-level hospital	Infectious diseases	Adult febrile illness	FLH18	Evaluation and management of fever in clinically unstable individuals using WHO IMAI guidelines, including empiric parenteral antimicrobials and antimalarial and resuscitative measures for septic shock	$84.39
First-level hospital	NCIP	CVD	FLH20	Management of acute coronary syndromes with aspirin, unfractionated heparin and generic thrombolytic (when indicated)	$268.17
First-level hospital	NCIP	CVD	FLH22	Management of acute coronary exacerbations of asthma and COPD using systemic steroids, inhaled beta-agonists and if indicated oral antibiotics and oxygen therapy	$50.90
First-level hospital	NCIP	CVD	FLH23	Medical management of acute heart failure	$387.77
First-level hospital	NCIP	Cancer	FLH24	Management of bowel obstruction	$167.20
First-level hospital	NCIP	Musculoskeletal	FLH25	Calcium and vitamin D supplementation for secondary prevention of osteoporosis	$147.97
First-level hospital	NCIP	Musculoskeletal	FLH26	Combination therapy, including low dose corticosteroids and generic disease modifying anti-rheumatic drugs (including methotrexate) for individuals with moderate to severe rheumatoid arthritis	$273.01
First-level hospital	NCIP	Congenital disorders	FLH27	In settings where sickle cell disease is a public health concern, universal new-born screening followed by standard prophylaxis against bacterial infections and malaria	$9.31
First-level hospital	NCIP	Congenital disorders	FLH28	In setting where specific single-gene disorders are a public health concern (for example thalassemia), retrospective identification of carriers plus prospective (premarital) screening and counselling to reduce rates of conception	$71.29
First-level hospital	NCIP	Injury	FLH30	Management of intoxication/ poisoning syndromes using widely available agents eg, charcoal, naloxone, bicarbonate, antivenin	$18.83
First-level hospital	Health services	Surgery	FLH31	Appendectomy	$172.07
First-level hospital	Health services	Surgery	FLH32	Assisted vaginal delivery using vacuum extraction or forceps	$166.39
First-level hospital	Health services	Surgery	FLH34	Colostomy (adult and paediatric)	$184.26
First-level hospital	Health services	Surgery	FLH35	Escharotomy or fasciotomy (adults)	$193.01
First-level hospital	Health services	Surgery	FLH36	Fracture reduction	$155.91
First-level hospital	Health services	Surgery	FLH37	Hernia repair including emergency surgery	$150.09
First-level hospital	Health services	Surgery	FLH38	Hysterectomy for uterine rupture or intractable postpartum haemorrhage	$204.52
First-level hospital	Health services	Surgery	FLH39	Irrigation and debridement of open fracture	$236.30
First-level hospital	Health services	Surgery	FLH40	Management of osteomyelitis, including surgical debridement	$254.37
First-level hospital	Health services	Surgery	FLH41a	Management of septic arthritis	$251.43
First-level hospital	Health services	Surgery	FLH41b	Placement of external fixation and use of traction for fractures	$214.64
First-level hospital	Health services	Surgery	FLH42	Relief of urinary obstruction by catheterization for fractures	$138.21
First-level hospital	Health services	Surgery	FLH43	Removal of gallbladder, including emergency surgery	$190.29
First-level hospital	Health services	Surgery	FLH44	Repair of perforations (for example perforated peptic ulcer, typhoid ileal perforation)	$240.26
First-level hospital	Health services	Surgery	FLH45	Resuscitation with advanced life support measures, including surgical airway	$50.13
First-level hospital	Health services	Surgery	FLH46	Basic skin grafting	$168.16
First-level hospital	Health services	Surgery	FLH48a	Trauma laparotomy	$221.35
First-level hospital	Health services	Surgery	FLH49	Trauma related amputations	$194.00
First-level hospital	Health services	Surgery	FLH50	Tube thoracostomy	$53.05
First-level hospital	Health services	Rehabilitation	FLH52	Compression therapy for amputations, burns, and vascular or lymphatic disorders	$5.43
First-level hospital	Health services	Rehabilitation	FLH53	Evaluation and acute management of swallowing dysfunction	$8.67
First-level hospital	Health services	Palliative care	FLH57	Prevention and relief of refractory suffering and acute pain related to surgery, serious injury or other serious, complex or life-limiting health problems	$1.40
First-level hospital	Health services	Pathology	FLH58	First level hospital pathology services	N/A
First-level hospital	RMNCH	Maternal and new-born health	HC6	Management of neonatal sepsis, pneumonia, and meningitis using injectable and oral antibiotics	$41.32
First-level hospital	RMNCH	Maternal and new-born health	HC10	Screening and management of diabetes in pregnancy (gestational diabetes or pre-existing type II diabetes)	$15.96
First-level hospital	RMNCH	Child health	HC13	Among all individuals who are known to be HIV+, immediate ART initiation with regular monitoring of viral load	$197.22
First-level hospital	Infectious diseases	HIV	HC19	For individuals testing positive for hepatitis B and C, assessment of treatment eligibility by trained providers followed by initiation and monitoring of ART when indicated	$188.69
First-level hospital	Infectious diseases	HIV	HC24	Hepatitis B vaccination for high-risk populations, including healthcare workers, IDU, MSM, household contacts and partners with multiple sex partners	$1.67
First-level hospital	Health services	Surgery	HC57b	Dental extraction	$13.93
First-level hospital	Health services	Palliative care	HC67	Expanded palliative care and pain control measures, including prevention and relief of all physical and psychological symptoms of suffering	$1.40
First-level hospital	RMNCH	Maternal and new-born health	RH1	Full supportive care for preterm new-borns	$24.14
First-level hospital	Health services	Surgery	RH14	Cataract extraction and insertion of intraocular lens	$151.84
Referral hospital	Health services	Surgery	FLH33	Craniotomy for trauma	$311.03
Referral hospital	Infectious diseases	TB	RH2	Specialized TB services, including management of MDR- and XDR-TB treatment failure and surgery for TB	$493.21
Referral hospital	Infectious diseases	Adult febrile illness	RH3	Management of refractory illness including etiological diagnosis at reference microbial laboratory	$673.43
Referral hospital	NCIP	CVD	RH4	Management of acute ventilator failure due to acute exacerbations of asthma and COPD	$30.11
Referral hospital	NCIP	CVD	RH5	Retinopathy screening via telemedicine, followed by treatment using laser photocoagulation	$1.72
Referral hospital	NCIP	CVD	RH6	Use of percutaneous coronary intervention for acute myocardial infarction where resources permit	$257.14
Referral hospital	NCIP	Cancer	RH7	Treatment of early-stage breast cancer with appropriate multimodal approaches (including generic chemotherapy) with curative intent for cases detected by clinical examination at health centres and first level hospitals	$1,304.04
Referral hospital	NCIP	Cancer	RH8	Treatment of early-stage colorectal cancer with appropriate multimodal approaches (including generic chemotherapy) with curative intent for cases detected by clinical examination at health centres and first level hospitals	$400.46
Referral hospital	NCIP	Cancer	RH9	Treatment of early-stage childhood cancers (such as Burkitt and Hodgkin lymphoma, acute lymphoblastic leukaemia, retinoblastoma and Wilms tumour) with curative intent in paediatric cancer units or hospitals	$1,477.76
Referral hospital	NCIP	Musculoskeletal	RH10	Elective surgical repair of common orthopaedic injuries (for example meniscal and ligamentous tears) in individuals with severe functional limitation	$239.90
Referral hospital	NCIP	Musculoskeletal	RH11	Urgent, definitive surgical management of orthopaedic injuries (for example open reduction and internal fixation)	$176.50
Referral hospital	NCIP	Congenital disorders	RH12	Repair of cleft lip and cleft palate	$515.11
Referral hospital	NCIP	Congenital disorders	RH13	Repair of club foot	$95.16
Referral hospital	Health services	Surgery	RH15	Repair of anorectal malformations and Hirschsprung’s disease	$230.76
Referral hospital	Health services	Surgery	RH16	Repair of obstetric fistula	$252.62
Referral hospital	Health services	Surgery	RH17	Ventriculoperitoneal shunt	$247.84
Referral hospital	Health services	Surgery	RH18	Surgery for trachomatous trichiasis	$136.67
Referral hospital	Health services	Pathology	RH19	Referral level hospital pathology services	N/A
Referral hospital	Health services	Pathology	RH20	Speciality pathology services	N/A

Abbreviations: DCP, Disease Control Priorities; iCCM, integrated community case management; WHO, World Health Organization; HiB, Haemophilus influenzae type b; BCG, Bacillus Calmette-Guéri; IRS, indoor residual spraying; HPV, human papillomavirus; STIs, sexually transmitted infections; TB, tuberculosis; WASH, water, sanitation and hygiene; G6PD, glucose-6-phosphate dehydrogenase; BEmONC, Basic Emergency Obstetric and Newborn Care; ADHD, attention deficit hyperactivity disorder; PLHIV, people living with HIV; IMAI, integrated management of adolescent and adult illness; CVD, cardiovascular disease; COPD, chronic obstructive pulmonary disease; ACEi, angiotensin-converting enzyme inhibitor; ARBs, angiotensin receptor blockers; IV, intravenous; CEmONC, Comprehensive Emergency Obstetric and Newborn Care; RMNCH, reproductive, maternal, new-born, child adolescent health/age related; ART, antiretroviral treatment; RH, reproductive health; NTDs, neglected tropical diseases; ECD, early childhood development; ARV, antiretroviral; IMCI, Integrated management of childhood illness; NCDIP, non-communicable disease and injury prevention; IUCDs, intra-uterine contraceptive devices; IDU, intravenous drug user; MSM, men who have sex with men; MDR, multidrug-resistant tuberculosis; XDR, extensively drug-resistant tuberculosis.

**Figure 2:**
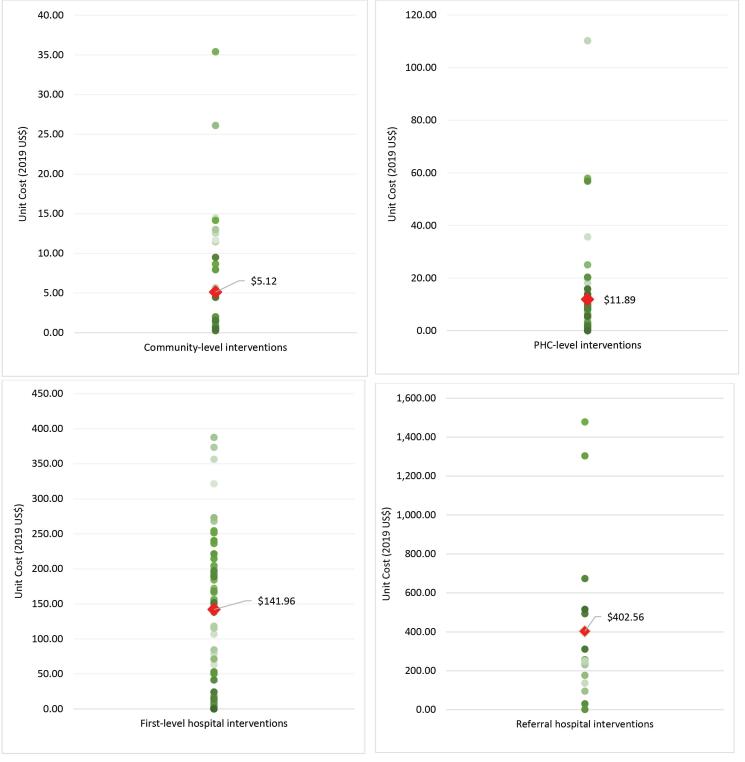
Distribution of Unit Costs and Mean Unit Cost by Platform (2019 US$).

**Figure 3:**
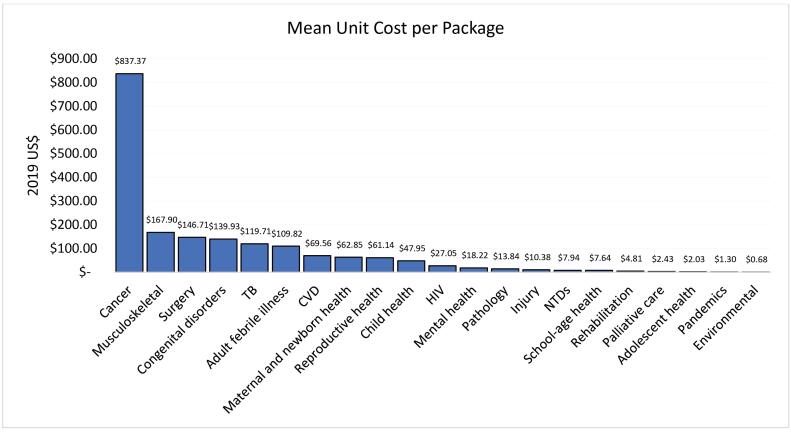
Mean Unit Cost Per Health Package (2019 US$). Abbreviations: TB, tuberculosis; CVD, cardiovascular disease; HIV, human immunodeficiency virus; NTDs, neglected tropical diseases.

 The mean unit costs varied increasingly by platform as seen in Figure 2: 2019 US$ 5.12 (0.27–35.40) in the community-based platform, 2019 US$ 11.89 (0.42–110.25) for health centre interventions, 2019 US$ 141.96 (1.16–387.77) in first-level hospitals, and 2019 US$ 402.56 (1.72–1477.76) in referral hospitals. The mean unit cost by disease area package (as defined by DCP3) also varied greatly. The packages with the highest mean unit cost per intervention were the cancer interventions (2019 US$ 837.37), musculoskeletal interventions (2019 US$ 167.90), and surgery (2019 US$ 146.71). Those with the lowest mean unit costs per intervention were environmental interventions (2019 US$ 0.68), pandemic-related interventions (2019 US$ 1.30) and adolescent health interventions (2019 US$ 2.03) (Figure 3).

**Figure 4:**
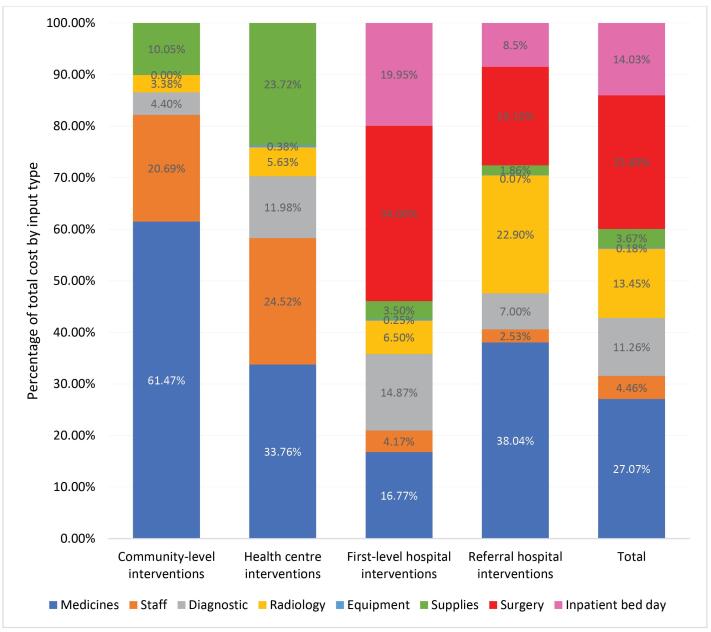
Cost Drivers by Platform.

 The overall largest cost drivers of unit costs overall were drug regimens and surgery-related costs, but the cost drivers also varied by service delivery platform. Staff costs were considerable for community and health centre interventions (21% and 25%, respectively), but less significant for first-level and referral hospitals (4% and 3%, respectively). Costs of medications made up a high percentage of total costs for community-level interventions (61%), referral hospitals (38%) and health centre interventions (34%). Surgery-related costs accounted for 34% and 19% of total costs in the first-level hospital and referral hospital interventions, respectively. Costs of inpatient bed-days made up 19% of total costs for first-level hospital interventions and 8% for referral hospitals. Supplies costs were higher for community-level and health centre interventions (10% and 24%, respectively) than for hospital-based interventions (3% at first-level hospitals and 2% at referral hospitals). Equipment costs were low in all platforms (<1%) (Figure 4).

**Figure 5:**
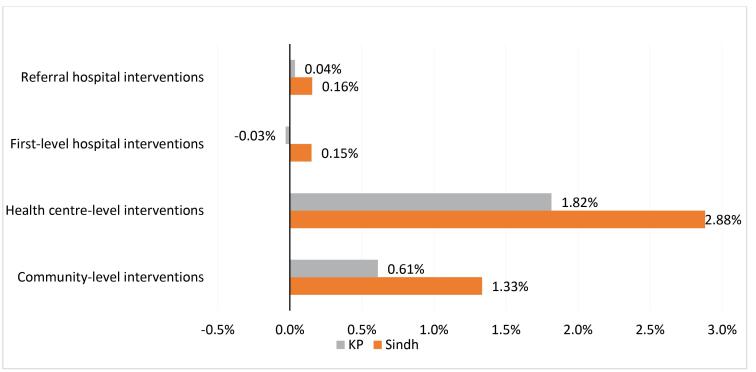
Tornado Diagram of Provincial Staff Salaries by Platform. Abbreviation: KP, Khyber Pakhtunkhwa.

**Figure 6:**
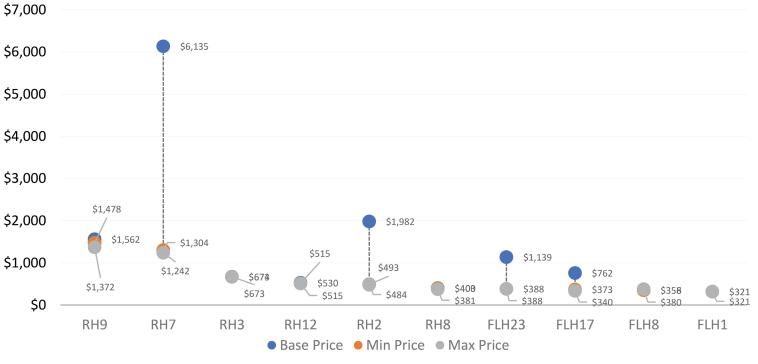
Sensitivity Analysis of Interventions With 10 Highest Unit Costs Using a range of Medicine Prices (2019 US$). Intervention codes (abbreviated): RH9: Treatment of early-stage childhood cancers. RH7: Treatment of early-stage breast cancer with appropriate multimodal approaches. RH3: Management of refractory illness. RH12: Repair of cleft lip and cleft palate. RH2: Specialized TB services, including management of MDR- and XDR-TB treatment failure and surgery. RH8: Treatment of early-stage colorectal cancer with appropriate multimodal approaches. FLH23: Medical management of acute heart failure. FLH17: Referral of cases of treatment failure for drug susceptibility testing; enrolment of those with MDR-TB for treatment. FLH8: Management of labour and delivery in high-risk women, including operative delivery (CEmONC). FLH1: Detection and management of foetal growth restriction.

 Sensitivity analysis showed that replacing federal health worker staff salaries with Khyber Pakhtunkhwa province salaries would alter the average unit costs by between a decrease of 0.03% for first-level hospitals and an increase of 1.82% at the health centre level. When using Sindh Province salaries, average unit costs increased by between 0.15% at the referral hospital level and 2.88% at the health centre level. Using alternative sources for medicine prices substantially changed unit costs for several of the highest unit cost interventions. Using the lowest price among the available medicine price sources led to moderate decreases in unit costs (by up to 7.13%, or 2019 US$ 62, for the treatment of early-stage childhood cancers). However, using the highest possible price available increased unit costs substantially in some interventions (by up to 370%, or 2019 US$ 4831, for the treatment of early-stage breast cancer) (Figures 5 and 6).

## Discussion

 We have presented the first comprehensive set of unit costs of health interventions in Pakistan estimated for the purposes of EPHS design. These unit costs were subsequently used during the priority setting process to calculate total costs, intervention-specific budget impact and to assess package affordability.^18^ We also demonstrate a rapid method which could be implemented in other countries where context-specific and comparable unit costs are required for EPHS design. In this discussion, we reflect on the process and outcome.

 Our costing method is appropriate for priority setting and is consistent with frameworks for fair decision-making, such as accountability for reasonableness.^26^ The dataset produced combines a large number of interventions, considers a comprehensive set of inputs per intervention and presents resource use and price data in a highly disaggregated manner and in a format that is highly accessible. Consequently, the data produced are transparent, relevant to the context and the decision problem faced by decision-makers and would be accessible during any revision or reversal processes. Further, a lack of sufficient and appropriate evidence is commonly cited as a barrier to decision-making around health investment and disinvestment; we address this gap by providing a broad set of country-specific cost estimates.

 During the planning stages, a number of publicly-available priority setting tools were reviewed, including, the Cost Revenue Analysis Tool Plus,^27^ the Health Interventions Prioritization Tool and the One Health Tool.^28^ While these tools have important added value for other parts of the priority setting process stakeholders, Pakistan opted for developing semi-automated user-friendly costing templates in Microsoft^®^ Excel for a number of reasons. Firstly, using a commonly available software that did not require extensive training was preferred in order to engage a broader set of stakeholders, both at national and provincial level, leaving the door open for regional adaptations or future package revisions. Secondly, a spreadsheet-based tool provided transparency in inputs and calculations which facilitate external validation of the data. Thirdly, it provided much-needed flexibility that allowed unit costs to be estimated for interventions with multiple service delivery configurations, different delivery platforms, as well as accounting for all intervention-specific fixed resources used. Excel-based tools have been used for health benefit package design in other LMIC settings.^29^

 HPSIU developed the service descriptions that served to determine resource use with a high-quality service in mind. This allowed for reflection on what constitutes a high-quality service and what aims the health system should strive towards. Although the process of cost calculation was carried out in six months, it was labour intensive. It required considerable input from several clinically trained members of staff at HPSIU (working full-time on this specific task), and, importantly, the aid of a wide range of TWG experts whose input enhanced the accuracy of the service descriptions. For a similar process to be successful elsewhere a number of factors will be required: a high level of technical expertise within the Ministry of Health, the ability to convene a wide range of actors and a high degree of political commitment. This process could also be expedited by having an available inventory of interventions, guidelines and resource needs to form the basis for Excel-based templates that could be adjusted locally. Such an inventory is currently under development at the World Health Organization (WHO) with the UHC Compendium.^30^

 Several price sources for different inputs, with different strengths and weaknesses, were identified. This prompted reflection on what constitutes desirable attributes of a price source when estimating costs for EPHS design. With the input of all stakeholders, we settled on three main criteria: (*i*) using recent prices, important in settings like Pakistan with high price fluctuations, (*ii*) using prices of purchase from the public sector, as the exercise was framed around public provision of services, and (*iii*) using prices reflective of the entire country, a difficult task in a highly heterogeneous setting as Pakistan. Although we found it helpful to keep these criteria in mind, we did not come to a resolution on how these three criteria should be traded off when one source did not possess all three attributes. More work needs to be done to develop a validated rapid process for selecting local prices to cost health benefit package interventions, as well as potentially finding adjustment factors between price sources. Further, estimates such as ours should be updated periodically to account for changes in prices due to medications going off patent, or reflecting a decrease in pricing due to government bulk purchasing or manufacturer rebates.^31^

 It is key to draw a distinction between priority setting and budgeting; our costs should not be directly translated into budgets without further adjustments. Our estimates will underestimate future financial expenditures in four ways. Firstly, our costs do not include indirect costs, above-service delivery costs or other overheads. Secondly, the approach does not capture health system inefficiencies or wastage. Thirdly, these estimates do not capture the costs incurred in carrying out initial consultations or diagnostics with patients whose conditions are not covered by the EPHS. Lastly, we calculated economic costs instead of financial costs. As a result, increased expenditure needed to purchase fixed resources at scale (eg, purchasing a dental chair for every health centre) will not be reflected. This highlights the importance, within the priority setting process, of thinking through issues of implementation, such as the bundling of interventions, which would enable more efficient use of common inputs.

 These under-estimations could be corrected by applying a mark-up, either a generic health system-wide one,^32^ or a context-specific one based on empirical evidence from primary costings, and by presenting economic and financial costs separately. Once the composition of the package has been agreed, further work will be needed to better calculate incremental financial expenditure. Such exercises have been carried out elsewhere in the South Asian sub-continent and may be helpful in Pakistan.^33^

 On the other hand, our unit costs may overestimate future provider expenditure. Firstly, certain goods presently procured as donations from international donors are accounted for even if no financial costs are incurred by the public sector. Secondly, the service delivery descriptions capture a delivery of high quality. Therefore, compared to current provision, some of our input costs may be higher than those of current interventions in order to compensate for both coverage and quality gaps. However, higher upfront costs for higher quality may paradoxically lead to lower overall costs; for instance, the United States National Academy of Medicine’s Crossing the Global Quality Chasm estimates an annual cost of $1.4-1.6 trillion in lost productivity due to poor-quality care.^34^ Nonetheless, correcting these overestimations may require reviewing costs as eligibility for donor funding schemes changes and as service delivery practices and guidelines are updated.

 Our unit costs were not highly sensitive to variations in staff salaries and are therefore, in that respect, likely to be generalisable across provinces. Some of the more costly interventions were highly sensitive to changes in medicine costs, particularly, early-stage cancer treatment and treatment for drug-resistant tuberculosis. Further work to understand province-specific medication procurement sources is important to produce accurate estimates.

 Our cost estimates were used to feed into the priority setting process, namely to understand budget impact of interventions, as well as to assess affordability within a budget constraint. Stakeholders reviewed these data, along with data on other decision criteria, such as cost-effectiveness and avoidable burden of disease, to make decisions on intervention inclusion and exclusion. This process, including the values that may have been prioritised and the trade-offs made, has been described in detail elsewhere.^18^

###  Limitations

 Our study is subject to a number of limitations. Firstly, while we attempted to develop a method that could be used across all EPHS cost calculations, we were not able to apply it consistently across inputs and intervention types. Our methods allowed for a rapid ingredients-based estimation of resource use. However, we found it difficult to apply this method when the inputs costed involved large numbers of components (generic inpatient bed-day and surgery costs) or complex diagnostic pathways and therefore had to rely on external estimates and prices from a study conducted in a large hospital in an urban setting, which may not be representative of resource use requirements in other areas of the country. Further, we used certain ballpark assumptions (eg, assuming the cost of furniture was 10% of building costs) which need to be further refined or tested. Secondly, we used expert opinion to fill gaps in certain inputs (eg, determining the length of time of an average consultation for a specific intervention). While we have explained why this method was preferable, and more feasible, than primary data collection, we acknowledge that eliciting information in such a way may introduce bias to our estimates. Thirdly, our analysis shows that average cost drivers vary by platform. However, the averages here presented are unweighted (in other words, they do not reflect the frequency of each intervention). Therefore, the average proportion of inputs per platform presented in this analysis is not reflective of the actual proportion of inputs that that will be needed for implementation per platform. Such calculations would ideally require combining unit costs with detailed geospatial data on effective coverage cascades detailing service need, use and quality, which was outside the scope of this analysis. Lastly, we were not able to adjust for within-country variation of resource use and prices (beyond our sensitivity analysis on staff salaries). Future exercises may want to use econometric or other methods to present a range of sub-national unit costs that better capture heterogeneity.^35^

## Conclusion

 We estimated 167 unit costs for the EPHS design process in Pakistan. We developed methods that allowed us to rapidly produce a large dataset of unit costs which were both context-specific and comparable. Further refinements to our methods are needed to better estimate costs of diagnostics, inpatient bed-days and generic surgeries for future application in Pakistan and for similar projects in other settings. A global inventory of interventions and their resource needs would greatly enhance efforts by countries wishing to develop their own costing tools in Microsoft^®^ Excel, rather than use programmed global ones. We have demonstrated that it is possible to estimate local costs, with expert validation and local acceptance, in rapid timeframes, rather than rely on extrapolated estimates from the limited global available of costs from other settings.

## Ethical issues

 Ethical approvals were obtained from the London School of Hygiene and Tropical Medicine (21247) and Aga Khan University (2019-1992-5190) and Ministry of Health clearance is being sought.

## Competing interests

 Authors declare that they have no competing interests.

## Data Availability Statement

 The full cost dataset is available upon request. Please contact Wajeeha Raza at raza.wajeeha@gmail.com.

## Funding

 This paper is part of a series of papers coordinated by the DCP3 Country Translation Project at the London School of Hygiene and Tropical Medicine, which is funded by the Bill & Melinda Gates Foundation [Grant OPP1201812]. The sponsor had no involvement in paper design; collection, analysis and interpretation of the data; and in the writing of the paper.

## Supplementary files


Supplementary file 1. Population-Level Interventions: Methods and Results.

Supplementary file 2. List of DCP3 Interventions Considered in the Priority-Setting Process but Not Costed.

Supplementary file 3. Further Details on Resource Use Estimation.

Supplementary file 4. Further Details on Price Sources Used.

Supplementary file 5. Alternative Sources of Unit Costs and Main Prices From the Peer-Reviewed Literature.
