# Supplementary material for: Costing Interventions for Developing an Essential Package of Health Services: Application of a Rapid Method and Results From Pakistan
Source: Int J Health Policy Manag. 2024 Jan 7;13:8006. doi: 10.34172/ijhpm.2023.8006 (PMC11607593; doi:10.34172/ijhpm.2023.8006)
Supplement: Supplementary file 1 — Population-Level Interventions: Methods and Results. [file ijhpm-13-8006-s001.pdf]

Article title: Costing Interventions for Developing an Essential Package of Health Services: Application of a Rapid Method and Results from Pakistan

Journal name: International Journal of Health Policy and Management (IJHPM)

Authors' information: Wajeeha Raza[1]*, Wahaj Zulfiqar[2], Mashal Murad Shah[3], Maryam Huda[3], Syeda Shehirbano Akhtar[2], Urooj Aqeel[2], Saira Kanwal[2], Muhammad Khalid[2], Raza Zaidi[2], Maarten Jansen[4], Nichola Kitson[5], Leon Bijlmakers[4], Sameen Siddiqi[3], Ala Alwan[6], Anna Vassall[5], Sergio Torres-Rueda[5]

[1]Centre for Health Economics, University of York, York, UK.

[2]Ministry of National Health Services, Regulations and Coordination, Islamabad, Pakistan. [3]Department of Community Health Sciences, Aga Khan University, Karachi, Pakistan. [4]Department of Health Evidence, Radboud Institute of Health Sciences, Radboud University Medical Centre, Nijmegen, The Netherlands.

[5]Department of Global Health & Development, London School of Hygiene and Tropical Medicine, London, UK.

[6]DCP3 Country Translation Project, London School of Hygiene and Tropical Medicine, London, UK.

***Correspondence to:** Wajeeha Raza; Email: raza.wajeeha@gmail.com

Citation: Raza W, Zulfiqar W, Shah MM, et al. Costing interventions for developing an essential package of health services: application of a rapid method and results from Pakistan. Int J Health Policy Manag. 2024;13:8006. doi:10.34172/ijhpm.2023.8006

Supplementary file 1. Population-Level Interventions: Methods and Results

Supplementary File 1 – Population-level interventions: methods and results

Population-based interventions were categorised into three groups: mass media interventions, interventions related to the development of national-level protocols, and high-level training and other exercises. These interventions have high fixed costs at the national level and take place above the service delivery level. A top-down costing approach was used.

To calculate costs, the HPSIU reviewed budget estimates of similar activities previously undertaken at the national level. We did not break down resource use by input as most expenditure was classified as contracted services (e.g., development of a television advertisement) and therefore difficult to disaggregate. Unit costs were estimated by dividing total national-level costs by an estimated population in need.

See Table A1.

Table A1: Population-based interventions: total costs, population in need and unit costs

**DCP code**	**Intervention**	**Total cost (2019 US$)**	**Population in need (description)**	**Population in need**	**Unit Cost (2019 US$)**
P1	Mass media messages concerning sexual and reproductive health and mental health for adolescents	$2,952,645.75	Population above the age of 10 years	160,556,777	$0.02
P2	Mass media messages concerning healthy eating or physical activity	$2,952,645.75	Population above the age of 10 years	160,556,777	$0.02
P3	Mass media messages concerning use of tobacco and alcohol	$2,952,645.75	Population above the age of 10 years	160,556,777	$0.02
P4	Mass media encouraging use of condoms, voluntary medical male circumcision and STI testing	$2,952,645.75	Population above the age of 10 years	160,556,777	$0.02
P6	Sustained integrated vector management for effective control of Chagas disease, visceral Leishmaniasis, dengue, chikungunya, CCHF and other	$2,986,536.07	Population above the age of 10 years	160,556,777	$0.02

DCP code	Intervention	Total cost (2019 US$)	Population in need (description)	Population in need	Unit Cost (2019 US$)
	nationally important causes of non-malarial fever vector borne NTDs				
P7	Conduct a comprehensive assessment of International Health Regulations (IHR) competencies using the Joint External Evaluation (JEE) tool	$57,121.28	Population above the age of 10 years	160,556,777	<$0.01
P8	Conduct simulation exercises and health worker training for outbreak events including outbreak investigation, contact tracing and emergency response	$154,838.71	Population above the age of 10 years	160,556,777	<$0.01
P9	Decentralize stocks of antiviral medications in order to reach at risk groups and disadvantaged populations	$175,750.95	Population at risk of HIV	1,254,507	$0.14
P10	Develop and implement a plan to ensure surge capacity in hospital beds, stockpiles of disinfectants, equipment for supportive care and personal protective equipment	$78,290,663.04	Population above the age of 10 years	160,556,777	$0.49
P11	Develop plans and legal authority for curtaining interactions between infected persons and un-infected population and implement and evaluate infection control measures in health facilities	$1,722,520.50	Population above the age of 10 years	160,556,777	$0.01
P13	Mass media messages concerning awareness on handwashing and health effects of household air pollution	$2,952,645.75	Population above the age of 10 years	160,556,777	$0.02
C25	Education campaign for the prevention of gender-based violence	$2,952,645.75	Population above the age of 10 years	160,556,777	$0.02
