# Supplementary material for: Costing Interventions for Developing an Essential Package of Health Services: Application of a Rapid Method and Results From Pakistan
Source: Int J Health Policy Manag. 2024 Jan 7;13:8006. doi: 10.34172/ijhpm.2023.8006 (PMC11607593; doi:10.34172/ijhpm.2023.8006)
Supplement: Supplementary file 2 — List of DCP3 Interventions Considered in the Priority-Setting Process but Not Costed. [file ijhpm-13-8006-s002.pdf]

Article title: Costing Interventions for Developing an Essential Package of Health Services: Application of a Rapid Method and Results from Pakistan

Journal name: International Journal of Health Policy and Management (IJHPM)

Authors' information: Wajeeha Raza[1]*, Wahaj Zulfiqar[2], Mashal Murad Shah[3], Maryam Huda[3], Syeda Shehirbano Akhtar[2], Urooj Aqeel[2], Saira Kanwal[2], Muhammad Khalid[2], Raza Zaidi[2], Maarten Jansen[4], Nichola Kitson[5], Leon Bijlmakers[4], Sameen Siddiqi[3], Ala Alwan[6], Anna Vassall[5], Sergio Torres-Rueda[5]

[1]Centre for Health Economics, University of York, York, UK.

[2]Ministry of National Health Services, Regulations and Coordination, Islamabad, Pakistan.

[3]Department of Community Health Sciences, Aga Khan University, Karachi, Pakistan.

[4]Department of Health Evidence, Radboud Institute of Health Sciences, Radboud University Medical Centre, Nijmegen, The Netherlands.

[5]Department of Global Health & Development, London School of Hygiene and Tropical Medicine, London, UK.

[6]DCP3 Country Translation Project, London School of Hygiene and Tropical Medicine, London, UK.

***Correspondence to:** Wajeeha Raza; Email: raza.wajeeha@gmail.com

Citation: Raza W, Zulfiqar W, Shah MM, et al. Costing interventions for developing an essential package of health services: application of a rapid method and results from Pakistan. Int J Health Policy Manag. 2024;13:8006. doi:10.34172/ijhpm.2023.8006

Supplementary file 2. List of DCP3 Interventions Considered in the Priority-Setting Process but Not Costed

File 2 – List of DCP3 interventions considered in the priority-setting process but not costed

FLH58 Specialty pathology services

RH19 Identify and refer patients with high risk

RH20 Prevention and relief of refractory suffering
