# Supplementary material for: Costing Interventions for Developing an Essential Package of Health Services: Application of a Rapid Method and Results From Pakistan
Source: Int J Health Policy Manag. 2024 Jan 7;13:8006. doi: 10.34172/ijhpm.2023.8006 (PMC11607593; doi:10.34172/ijhpm.2023.8006)
Supplement: Supplementary file 3 — Further Details on Resource Use Estimation. [file ijhpm-13-8006-s003.pdf]

Article title: Costing Interventions for Developing an Essential Package of Health Services: Application of a Rapid Method and Results from Pakistan

Journal name: International Journal of Health Policy and Management (IJHPM)

Authors' information: Wajeeha Raza[1]*, Wahaj Zulfiqar[2], Mashal Murad Shah[3], Maryam Huda[3], Syeda Shehirbano Akhtar[2], Urooj Aqeel[2], Saira Kanwal[2], Muhammad Khalid[2], Raza Zaidi[2], Maarten Jansen[4], Nichola Kitson[5], Leon Bijlmakers[4], Sameen Siddiqi[3], Ala Alwan[6], Anna Vassall[5], Sergio Torres-Rueda[5]

[1]Centre for Health Economics, University of York, York, UK.

[2]Ministry of National Health Services, Regulations and Coordination, Islamabad, Pakistan.
[3]Department of Community Health Sciences, Aga Khan University, Karachi, Pakistan.
[4]Department of Health Evidence, Radboud Institute of Health Sciences, Radboud University Medical Centre, Nijmegen, The Netherlands.

[5]Department of Global Health & Development, London School of Hygiene and Tropical Medicine, London, UK.

[6]DCP3 Country Translation Project, London School of Hygiene and Tropical Medicine, London, UK.

***Correspondence to:** Wajeeha Raza; Email: raza.wajeeha@gmail.com

Citation: Raza W, Zulfiqar W, Shah MM, et al. Costing interventions for developing an essential package of health services: application of a rapid method and results from Pakistan. Int J Health Policy Manag. 2024;13:8006. doi:10.34172/ijhpm.2023.8006

Supplementary file 3. Further Details on Resource Use Estimation

Staff requirements were described in terms of staff type and duration of direct contact with the patient. For activities carried out at the community level, a standard seven minutes per patient were added to account for average health worker transportation time between households, as per HPSIU estimates. Community health workers in Pakistan include lady health workers, who work in rural areas and travel by foot between houses. The lady health worker project was developed to allow health workers hired from the geographical area to travel by foot to each household. Each lady health worker only serves a population where she can travel easily by foot and therefore the distances that they travel are limited.

Certain inputs, such as the length of consultations or the average number of days in hospital for inpatient services, were estimated and/or validated through expert opinion. A group of 6-8 clinicians from the Aga Khan University Hospital in Karachi participated in working groups where they discussed resource use descriptions. They were asked to discuss among themselves and agree on a base case estimate.

Drug regimens were described by including the medication type, dose, frequency of use and duration of treatment. For some interventions, multiple drug regimens were described depending on the target population. In these cases, we used a weighted average (based on the percentage of patients using each regimen) to calculate costs. The types and total number of diagnostic procedures were specified, as were other supplies used. Equipment resource use was quantified by the number of minutes used per intervention.

For outpatient services, building costs (space, utilities and furniture) were quantified by estimating the rental costs of the room per minute using procurement documents for the Islamabad Capital Territory region [1]. These costs were then multiplied by the number of minutes of staff time required for each intervention.

Resource use for inpatient bed-days and surgeries were calculated using peer-reviewed literature as a protocol-based cost would not have been appropriate given the large quantities of supplies and equipment generally used. A literature search was carried out and Khan et al.'s (2017) activity-based hospital costing study was deemed to be the only appropriate study, as it contained disaggregated hospital inputs and costs in a Pakistani setting, and as the methodology was clearly outlined and in line with our approach [2]. The paper reports the results of a knee replacement surgery in a Karachi hospital broken down by phase of care. Raw data was shared by the authors and was further disaggregated. We reviewed cost inputs, including ancillary staff, for both the inpatient ward day and the surgery, removing inputs that were knee surgery-specific in order to arrive at a generic list of resources applicable to inpatient bed-days and surgeries across disease areas. These standard quantities of resources used for both bed-days and surgeries were used for all DCP3 interventions. Building costs were included in the study. Daily services such as laundry and food catering [3, 4] , were added. Additional equipment or supplies required

beyond what was contained in the standard package were also added to the intervention-specific service descriptions.

Due to feasibility issues, MNHSR&C changed the delivery platform specified in 23 DCP3 interventions to better suit the national context; interventions were costed assuming resource use in the platform in which they would be delivered in Pakistan. See Table A2.

Table A2: Interventions costed assuming implementation in platforms different from those in DCP3

DCP platform	Pakistan platform	Intervention Code DCP	Intervention Name
Health centre	Community	HC4a	Condoms and hormonal contraceptives
Health centre	Community	HC5a	Counselling on kangaroo care for new-borns
Health centre	Community	HC9a	Screening of hypertensive disorders in pregnancy
Health centre	Community	HC28	Screening for HIV in all individuals with a diagnosis of active TB; if HIV infection is present, start (or refer for) ARV treatment and HIV care
Health centre	Community	HC66	Psychosocial support and counselling services for individuals with serious, complex, or life-limiting health problems and their caregivers
Population	Community	P5	Systematic identification of individuals with TB symptoms among high-risk groups and linkage to care ("active case finding")
Community	Health centre	C3c	Management of labour and delivery in low-risk women by skilled attendant
Community	Health centre	C3d	Basic neonatal resuscitation following delivery
Community	Health centre	C33	For malaria due to P. vivax, test for G6PD deficiency; if normal, add chloroquine or chloroquine plus 14-day course of primaquine
Community	Health centre	C5	Tetanus toxoid immunization among schoolchildren and among women attending antenatal care
Community	Health centre	C27b	Provision of iron and folic acid supplementation to pregnant women, and provision of food or caloric supplementation to pregnant women in food-insecure households

**DCP platform**	**Pakistan platform**	**Intervention Code DCP**	**Intervention Name**
Community	Health centre	C53b	ECD rehabilitation interventions
Community	FLH	C50	Parent training of high-risk families, including nurse home visitation for child maltreatment
Health centre	FLH	HC6	Management of neonatal sepsis, pneumonia, and meningitis<br>using injectable and oral antibiotics
Health centre	FLH	HC10	Screening and management of diabetes in pregnancy (gestational diabetes or pre-existing type II diabetes)
Health centre	FLH	HC13	Among all individuals who are known to be HIV+, immediate ART initiation with regular monitoring of viral load
Health centre	FLH	HC19	For individuals testing positive for hepatitis B and C, assessment of treatment eligibility by trained providers followed by initiation and monitoring of ART when indicated
Health centre	FLH	HC24	Hepatitis B vaccination for high-risk populations, including healthcare workers, IDU, MSM, household contacts and partners with multiple sex partners
Health centre	FLH	HC57b	Dental Extraction
Health centre	FLH	HC67	Expanded palliative care and pain control measures, including prevention and relief of all physical and psychological symptoms of suffering
Referral hospital	FLH	RH1	Full supportive care for preterm new-borns
Referral hospital	FLH	RH14	Cataract Extraction and Insertion of Intraocular Lens
First-level hospital	RH	FLH33	Craniotomy for Trauma

References

1. Ministry of National Health Services Regulation and Coordination, *Islamabad Capital Territory Health Infrastructure 2020-2021 PC-1.* 2020.
2. Khan, R.M., et al., *Time-driven activity-based costing of total knee replacements in Karachi, Pakistan.* BMJ Open, 2019. **9**(5): p. e025258.
3. Ministry of Industries and Production, *Pre-feasibility Study: Laundry and dry cleaning.* .
4. Sagheer U., et al., *Cost of establishing and maintaining a bed in a tertiary care hospital in Pakistan*, in *Leadership in Health Services*. 2000.
