# Supplementary material for: Costing Interventions for Developing an Essential Package of Health Services: Application of a Rapid Method and Results From Pakistan
Source: Int J Health Policy Manag. 2024 Jan 7;13:8006. doi: 10.34172/ijhpm.2023.8006 (PMC11607593; doi:10.34172/ijhpm.2023.8006)
Supplement: Supplementary file 4 — Further Details on Price Sources Used. [file ijhpm-13-8006-s004.pdf]

Article title: Costing Interventions for Developing an Essential Package of Health Services: Application of a Rapid Method and Results from Pakistan

Journal name: International Journal of Health Policy and Management (IJHPM)

Authors' information: Wajeeha Raza[1]*, Wahaj Zulfiqar[2], Mashal Murad Shah[3], Maryam Huda[3], Syeda Shehirbano Akhtar[2], Urooj Aqeel[2], Saira Kanwal[2], Muhammad Khalid[2], Raza Zaidi[2], Maarten Jansen[4], Nichola Kitson[5], Leon Bijlmakers[4], Sameen Siddiqi[3], Ala Alwan[6], Anna Vassall[5], Sergio Torres-Rueda[5]

[1]Centre for Health Economics, University of York, York, UK.

[2]Ministry of National Health Services, Regulations and Coordination, Islamabad, Pakistan.
[3]Department of Community Health Sciences, Aga Khan University, Karachi, Pakistan.
[4]Department of Health Evidence, Radboud Institute of Health Sciences, Radboud University Medical Centre, Nijmegen, The Netherlands.

[5]Department of Global Health & Development, London School of Hygiene and Tropical Medicine, London, UK.

[6]DCP3 Country Translation Project, London School of Hygiene and Tropical Medicine, London, UK.

***Correspondence to:** Wajeeha Raza; Email: raza.wajeeha@gmail.com

Citation: Raza W, Zulfiqar W, Shah MM, et al. Costing interventions for developing an essential package of health services: application of a rapid method and results from Pakistan. Int J Health Policy Manag. 2024;13:8006. doi:10.34172/ijhpm.2023.8006

Supplementary file 4. Further Details on Price Sources Used

Federal-level healthcare worker pay scales were used to determine average staff time pricing per health worker cadre [1]. This source was selected as it was both recent and from the public sector. When activities could be carried out by multiple members of staff (e.g., nurse or lady health worker), we used salaries for each type of worker, costing each staff configuration as a separate intervention, and presented an average unit cost weighting options equally.

There were a number of sources available for the price of medications. No source contained all medications used. The primary source of price data used was the Sindh Health Department Procurement Price list of 2018-19 [2]. This was found to be the most appropriate price source

since it was both recent and listed public sector prices. If a price was unavailable in the Sindh procurement list, the Federal Wholesale Price List for Generic Medicines was used as a second option [3]. The third option was the list of procurement purchasing prices from a public-private partnership under the Medical Emergency Resilience Fund 2019-2020 [4]. Private sector wholesale market prices were used as a fourth and final option [5].

The percentage of medication prices sourced from the different lists is as follows:

**Cluster A**	
Sindh Health Department Procurement Price list of 2018-19	39.5%
Federal Wholesale Price List	34.9%
Medical Emergency Resilience Fund 2019-2020	9.3%
Private Sector Wholesale Market Prices	11.6%
Private International Market Prices	4.7%
**Cluster B**	
Sindh Health Department Procurement Price list of 2018-19	34.4%
Federal Wholesale Price List	62.5%
Medical Emergency Resilience Fund 2019-2020	3.1%
**Cluster C**	
Federal Wholesale Price List	57.6%
Medical Emergency Resilience Fund 2019-2020	36.4%
Private Sector Wholesale Market Prices	6%
**Cluster D**	
Sindh Health Department Procurement Price list of 2018-19	9.8%
Federal Wholesale Price List	53.7%
Medical Emergency Resilience Fund 2019-2020	4.8%
Private Sector Wholesale Market Prices	31.7%

A price source hierarchy was also established for supplies and equipment. The first choice of source was procurement prices from the Medical Emergency Resilience Fund 2019-2020 [4]. When price data were unavailable, we used private sector market prices [5]. Equipment costs were treated as capital costs and were annuitized using a 3% discount rate. An average useful life for each piece of equipment was estimated with the input of HPSIU.

The percentage of equipment prices sourced from the different lists is as follows:

**Cluster A**	
Medical Emergency Resilience Fund 2019-2020	96.3%
Private International Market Prices	3.7%

Cluster B	
Medical Emergency Resilience Fund 2019-2020	100%
**Cluster C**	
Medical Emergency Resilience Fund 2019-2020	100%
**Cluster D**	
Medical Emergency Resilience Fund 2019-2020	63.4%
Private Sector Wholesale Market Prices	36.6%

Physical space prices were calculated by using price data from budget documents from the Federal government [6]. We obtained the estimated price of utilities per consultation from a costing study carried out in Khyber Pakhtunkhwa Province [7]. A generic cost of furniture was added and assumed at 10% of the cost of space.

We were unable to construct diagnostic and radiology costs through an ingredients-based approach ourselves due to time constraints and the complexity of supplies and equipment used. We resorted to available literature and market prices. We assessed strengths and weaknesses of different price and cost sources. We used the 'Costing and Pricing of Services in Private Hospitals of Lahore: Summary Report' as our primary source as it also used an ingredients-based approach which is consistent with our methodology [8]. If costs were unavailable, we used user fees from the Pakistan Institute of Medical Sciences [9] as a secondary option. We further used prices charged by private laboratories: Chugtai Labs user fees [10] were the third option and fees charged by the Aga Khan University Hospital (AKU) in Karachi [11] were the fourth and final option.

Prices for the generic surgery and ward day unit cost were obtained from the same sources as the resource use data [12, 13].

References

1. Ministry of National Health Services, R.a.C., *Federal Pay Scales for Health Workers Islamabad Capital Territory*. 2019.
2. Government of Sindh, H.D., *Procurement of Drugs, Medicines, Surgical and Disposable Items, Xray files, Chemicals & Allied Items Under Rate Contract Basis During the Year*. 2019.

3. Ministry of National Health Services Regulation and Coordination, *Wholesale market prices for generic medicines*. 2019.
4. Medical Emergency Resilience Fund, *Procurement Prices*. 2019.
5. Online Drug Information System. *Pharma professional services Pakistan*. 2019; Available from: http://www.druginfosys.com/.
6. Ministry of National Health Services Regulation and Coordination, *Islamabad Capital Territory Health Infrastructure 2020-2021 PC-1*. 2020.
7. Ministry of National Health Services Regulation & Coordination, *Khyber Pakhtunkhwa Province Hospital Costing*.
8. Punjab Healthcare Commission, *Costing and pricing of services in private hospitals of Lahore*. 2018.
9. Pakistan Institute of Medical Sciences, *Pathology Department Price List (payment)*. 2019.
10. Chugtai Labs, *Diagnostic user fees*. 2019.
11. Aga Khan University Hospital, *Diagnostic User Fees*. 2019.
12. Khan, R.M., et al., *Time-driven activity-based costing of total knee replacements in Karachi, Pakistan*. BMJ Open, 2019. **9**(5): p. e025258.
13. Sagheer U., et al., *Cost of establishing and maintaining a bed in a tertiary care hospital in Pakistan*, in *Leadership in Health Services*. 2000.
