# Supplementary material for: Costing Interventions for Developing an Essential Package of Health Services: Application of a Rapid Method and Results From Pakistan
Source: Int J Health Policy Manag. 2024 Jan 7;13:8006. doi: 10.34172/ijhpm.2023.8006 (PMC11607593; doi:10.34172/ijhpm.2023.8006)
Supplement: Supplementary file 5 — Alternative Sources of Unit Costs and Main Prices From the Peer-Reviewed Literature. [file ijhpm-13-8006-s005.pdf]

Article title: Costing Interventions for Developing an Essential Package of Health Services: Application of a Rapid Method and Results from Pakistan

Journal name: International Journal of Health Policy and Management (IJHPM)

Authors' information: Wajeeha Raza[1]*, Wahaj Zulfiqar[2], Mashal Murad Shah[3], Maryam Huda[3], Syeda Shehirbano Akhtar[2], Urooj Aqeel[2], Saira Kanwal[2], Muhammad Khalid[2], Raza Zaidi[2], Maarten Jansen[4], Nichola Kitson[5], Leon Bijlmakers[4], Sameen Siddiqi[3], Ala Alwan[6], Anna Vassall[5], Sergio Torres-Rueda[5]

[1]Centre for Health Economics, University of York, York, UK.

[2]Ministry of National Health Services, Regulations and Coordination, Islamabad, Pakistan.
[3]Department of Community Health Sciences, Aga Khan University, Karachi, Pakistan.
[4]Department of Health Evidence, Radboud Institute of Health Sciences, Radboud University Medical Centre, Nijmegen, The Netherlands.

[5]Department of Global Health & Development, London School of Hygiene and Tropical Medicine, London, UK.

[6]DCP3 Country Translation Project, London School of Hygiene and Tropical Medicine, London, UK.

***Correspondence to:** Wajeeha Raza; Email: raza.wajeeha@gmail.com

Citation: Raza W, Zulfiqar W, Shah MM, et al. Costing interventions for developing an essential package of health services: application of a rapid method and results from Pakistan. Int J Health Policy Manag. 2024;13:8006. doi:10.34172/ijhpm.2023.8006

Supplementary file 5. Alternative Sources of Unit Costs and Main Prices From the Peer-Reviewed Literature.

Intervention	Type of cost	Cost (USD)	Reason for using cost from literature	Source
HC25. Provision of voluntary medical male circumcision in settings with high prevalence of HIV	Cost of a VMMC surgical set	$23.00	Many implements involved and easier to use literature. Many costings have been done in this area already.	Larson et al. (2015) (15)

Intervention	Type of cost	Cost (USD)	Reason for using cost from literature	Source
HC13. Among all individuals who are known to be HIV+, immediate ART initiation with regular monitoring of viral load	Cost of viral load test	$20.50	Costs only found in AKU (prices). Prices considered to be many times higher than international estimates	Freedberg et al (2018) (16)
HC13. Among all individuals who are known to be HIV+, immediate ART initiation with regular monitoring of viral load	Cost of CD4 test	$3.40	Costs only found in AKU (prices). Prices considered to be many times higher than international estimates	Freedberg et al (2018) (16)
HC13. Among all individuals who are known to be HIV+, immediate ART initiation with regular monitoring of viral load	Cost of first-line treatment for adults (yearly)	$96.00	Prices across all price lists led to total costs that were 10-12 times higher than the average costs for LMICs. We assume these lists did not take into account tiered pricing used by governments.	Freedberg et al (2018) (16)
HC13. Among all individuals who are known to be HIV+, immediate ART initiation with regular monitoring of viral load	Cost of second-line treatment for adults (yearly)	$260.00	Prices across all price lists led to total costs that were 10-12 times higher than the average costs for LMICs. We assume these lists did not take into account tiered pricing used by governments.	Freedberg et al (2018) (16)
HC13. Among all individuals who are known to be HIV+, immediate ART	Cost of first-line treatment for	$96.00	Prices across all price lists led to total costs that were 10-12 times higher than the	Freedberg et al (2018) (16)

Intervention	Type of cost	Cost (USD)	Reason for using cost from literature	Source
initiation with regular monitoring of viral load	children (yearly)		average costs for LMICs. We assume these lists did not take into account tiered pricing used by governments. Note that we have not differentiated prices between adult and children's dosages.	
HC13. Among all individuals who are known to be HIV+, immediate ART initiation with regular monitoring of viral load	Cost of second-line treatment for children (yearly)	$260.00	Prices across all price lists led to total costs that were 10-12 times higher than the average costs for LMICs. We assume these lists did not take into account tiered pricing used by governments. Note that we have not differentiated prices between adult and children's dosages.	Freedberg et al (2018) (16)
FLH17. Referral of cases of treatment failure for drug susceptibility testing; enrolment of those with MDR-TB for treatment per WHO guidelines (either short- or long-term regimen)	Cost of a GeneXpert test	$24.42	Costs only found in AKU (prices). Prices considered to be many times higher than international estimates	Vassall et al. (2017) (17)
RH13. Repair of clubfoot	Cost of a unilateral club foot brace	$90.00	We could not find a local cost. This cost represents the	Grimes et al. (2016) (18)

Intervention	Type of cost	Cost (USD)	Reason for using cost from literature	Source
			main part of the intervention.	

Note: During the deliberation process we presented these cost estimates in the currency year in which they were found in the literature, mentioning it to stakeholders and therefore asking them to understand there was greater uncertainty around these specific estimates. We decided against adjusting these inputs according to inflation because we did not want to provide a potentially false sense of equivalence. There are a number of reasons why estimates sourced elsewhere could be misleading. For example, medications go from patented to generic after a certain number of years, a change which would not be captured by inflation indices. Further, these difficulties are compounded by transferability concerns, such as country-specific manufacturer rebates or bulk purchasing.

References

1. Khan RM, Albutt K, Qureshi MA, Ansari Z, Drevin G, Mukhopadhyay S, et al. Time-driven activity-based costing of total knee replacements in Karachi, Pakistan. BMJ Open. 2019;9(5):e025258.
2. Sagheer U., Kielmann A., Mumtaz Z, S. S. Cost of establishing and maintaining a bed in a tertiary care hospital in Pakistan. 2000.
3. Ministry of Industries and Production. Pre-feasibility Study.
4. Ministry of National Health Services RaC. Federal Pay Scales for Health Workers Islamabad Capital Territory. 2019.
5. Government of Sindh HD. Procurement of Drugs, Medicines, Surgical and Disposable Items, Xray files, Chemicals & Allied Items Under Rate Contract Basis During the Year. 2019.
6. Ministry of National Health Services Regulation and Coordination. Wholesale market prices for generic medicines. 2019.
7. Medical Emergency Resilience Fund. Procurement Prices. 2019.
8. Online Drug Information System. Pharma professional services Pakistan 2019 [Available from: http://www.druginfosys.com/.
9. Ministry of National Health Services Regulation and Coordination. Islamabad Capital Territory Health Infrastructure 2020-2021 PC-1. 2020.
10. Ministry of National Health Services Regulation & Coordination. Khyber Pakhtunkhwa Province Hospital Costing.
11. Punjab Healthcare Commission. Costing and pricing of services in private hospitals of Lahore. 2018.
12. Pakistan Institute of Medical Sciences. Pathology Department Price List (payment). 2019.
13. Chugtai Labs. Diagnostic user fees. 2019.
14. Aga Khan University Hospital. Diagnostic User Fees. 2019.

15. Larson B, Tindikahwa A, Mwidu G, Kibuuka H, Magala F. How much does it cost to improve access to voluntary medical male circumcision among high-risk, low-income communities in Uganda? PLoS One. 2015;10(3):e0119484.
16. Freedberg KA, Kumarasamy N, Borre ED, Ross EL, Mayer KH, Losina E, et al. Clinical Benefits and Cost-Effectiveness of Laboratory Monitoring Strategies to Guide Antiretroviral Treatment Switching in India. AIDS Res Hum Retroviruses. 2018;34(6):486-97.
17. Vassall A, Siapka M, Foster N, Cunnama L, Ramma L, Fielding K, et al. Cost-effectiveness of Xpert MTB/RIF for tuberculosis diagnosis in South Africa: a real-world cost analysis and economic evaluation. Lancet Glob Health. 2017;5(7):e710-e9.
18. Grimes CE, Holmer H, Maraka J, Ayana B, Hansen L, Lavy CBD. Cost-effectiveness of club-foot treatment in low-income and middle-income countries by the Ponseti method. BMJ Glob Health. 2016;1(1):e000023.
